# 53 years old is a reasonable cut-off value to define young and old patients in clear cell renal cell carcinoma: a study based on TCGA and SEER database

**DOI:** 10.1186/s12885-021-08376-5

**Published:** 2021-05-29

**Authors:** Fucai Tang, Zechao Lu, Chengwu He, Hanbin Zhang, Weijia Wu, Zhaohui He

**Affiliations:** 1 Department of Urology, The Eighth Affiliated Hospital, Sun Yat-sen University, Shennan Zhong Road #3025, Futian District, Shenzhen, 518033 Guangdong China; 2 The First Clinical College of Guangzhou Medical University, Guangzhou, 511436 China; 3 The Second Clinical College of Guangzhou Medical University, Guangzhou, 511436 China

**Keywords:** Age, Age-related genes, Clear cell renal cell carcinoma, The Cancer genome atlas, Surveillance epidemiology and end results

## Abstract

**Background:**

The objectives of this study were to screen out cut-off age value and age-related differentially expressed genes (DEGs) in clear cell renal cell carcinoma (CCRCC) from Surveillance Epidemiology and End Results (SEER) database and The Cancer Genome Atlas (TCGA) database.

**Methods:**

We selected 45,974 CCRCC patients from SEER and 530 RNA-seq data from TCGA database. The age cut-off value was defined using the X-tile program. Propensity score matching (PSM) was used to balance the differences between young and old groups. Hazard ratio (HR) was applied to evaluate prognostic risk of age in different subgroups. Age-related DEGs were identified via RNA-seq data. Survival analysis was used to assess the relationship between DEGs and prognosis.

**Results:**

In this study, we divided the patients into young (*n* = 14,276) and old (*n* = 31,698) subgroups according to cut-off value (age = 53). Age > 53 years was indicated as independent risk factor for overall survival (OS) and cancer specific survival (CSS) of CCRCC before and after PSM. The prognosis of old group was worse than that in young group. Eleven gene were differential expression between the younger and older groups in CCRCC. The expression levels of PLA2G2A and SIX2 were related to prognosis of the elderly.

**Conclusion:**

Fifty-three years old was cut-off value in CCRCC. The prognosis of the elderly was worse than young people. It remind clinicians that more attention and better treatment should be given to CCRCC patients who are over 53 years old. PLA2G2A and SIX2 were age-related differential genes which might play an important role in the poor prognosis of elderly CCRCC patients.

## Background

Over the past two decades, the incidence of renal cell carcinoma (RCC) at every stages was increased and this situation resulted in a steady increase in mortality per unit of population [1]. It is estimated that 65,340 Americans will be diagnosed with RCC, and 14,970 Americans will die of this cancer in 2018. RCC comprises about 3.8% of all new cancer. And the median age of RCC patient is 64 ages old. Clear cell renal cell carcinoma (CCRCC) is the most common subtype of RCC, it accounts for about 80% of RCC [2]. Age has prognostic significance in many solid cancers, and one of renal cancer known risk factors is age [3, 4]. RCC shows a more favorable prognosis in young patiens, which may be due to the lower state of diagnosis [5]. In addition, age can influence the structural and molecular properties of the tumor vasculature in CCRCC by comparing the vascular properties of patients who over the age of 65 and under 65 years old [6]. Furthermore, expression levels of Piwil 1 mRNA in patients who under 64 years old are higher than that in older people (> 64 years old). But there still is no optimal age cut off value to define elderly and young people in CCRCC. Therefore, we determined the optimal cut-off value for age analyzing the clinical data SEER database, and explored differentially expressed genes (DEGs) between older and younger people of CCRCC by analyzing RNA-seq data from TCGA in present study.

## Methods

### Study population from SEER

SEER Stat software (version 8.3.5) was used to download CCRCC clinical data from the National Cancer Institute’s Surveillance, Epidemiology, and End Results (SEER) database. The downloaded data included: patient ID, the year and age at the time of diagnosis, sex, race, histological type, survival time, tumor size, marital status, grade, SEER historic stage A, and cause of death.

CCRCC patients were selected according to the following criteria: (1) site record International Classification of Diseases for Oncology, Third Revision (ICD-O-3) was C649; (2) histological type was 8310/3; (3) the year at time of diagnosis was 1988–2014. (4) CCRCC was primary tumor. The exclusion criteria were listed as following: (1) patients without race and gender information; (2) patients whose tumor size, survival time and other clinical information we need in this study were unknown.

### Variable declaration

Race was defined as white, black and other. Marital was divided into Single/Other, and married. Tumor size was divided into less than 4 cm, 4 cm to 7 cm, and greater than 7 cm. Grade was grouped as I, II, III, IV. Laterality was divided into left and right. The SEER historic stage options included localized, regional and distant. And the chemotherapy, radiotherapy were divided into yes or no.

### Cut off age in CCRCC

X-tile is a useful tool for biomarker assessment and outcome-based cut-point optimization (http://www.tissuearray.org/rimmlab/). The “x tile plot” can provide a single, global assessment of every possible way by dividing a population into low- and high-level marker expression [7]. The grouping strategy of the X-tile program includes trying to use each number between the retrieved count ranges as a critical value, then, using this number as a cut-off value to calculate the χ2 score and *P* value. We used X-tile plots to assessed all possible age cutoff value, and the survival at every age cutoff value was computed by the log rank test. Then the most appropriate cut-off value was selected which had the highest χ2 value.

### RNA-seq analysis of CCRCC from TCGA

The RNA sequencing and clinical information of CCRCC were download from TCGA database. We used these RNA-seq data for DEGs screening between younger and older group by Limma package (adjusted *p* value < 0.05 and | log2 fold change (FC) | ≥1). Then, we extracted clinical data from older adults (> 53 years), including survival time and survival status. We selected the DEGs from the small to large false discovery rate (FDR). And the DEGs was for survival analysis. The differentially expression levels of DEGs in these old patients were obtained. The median of gene expression was used to classify low and high group. Log rank test was used to compare statistically significant differences between high and low expression groups.

### Statistical analysis

We divided the patients into young and old groups according to the X-tile’s best cut-off value. Chi-square test was used to compare the differences in the distribution of variables between younger and older group. We calculated the overall survival (OS) and cancer specific survival (CSS). In the CSS calculation, the cause of death for other reasons was defined as censorship. Propensity score matching (PSM) used logistic regression included relevant variables of sex, race, marital status, size, grade, SEER historic stage A, radiation and chemotherapy to balance the baseline differences between the younger and older groups. The OS and CSS Survival curves were generated using the Kaplane-Meier method. And univariate and multivariate analysis Cox regression models were applied to adjust prognostic variables. The cases were stratified according to the relevant variables. Hazard Ratio (HR) of the CSS was calculated according to the age. When the 2-sided *P* value was < 0.05, the differences were considered statistically significant. The SPSS 24.0 and R 3.4.3 were used to conduct statistical analysis and DEGs screening.

## Result

### 53 was the age cut-off value and baseline characteristics

**Fig. 1:**
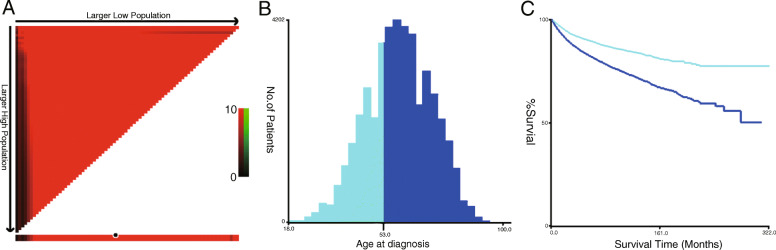
X-Tile analysis of survival data from the Surveillance, Epidemiology, and End Results (SEER) registry. X-Tile analysis was performed using patient data from the SEER registry. The plot showed the χ2 log-rank values produced when dividing the cohort with two cut-points, producing low and high subsets. The X-axis represented all potential cut-off point from low to high that defined a low subset, while the Y-axis represented the high to low cut-off points that defined a high subset. The arrows indicated the direction in which the size of the low subset (X-axis) and the high subset (Y-axis) increased. **A** The red coloration of cut-points indicates an negative correlation with survival, whereas green coloration represents positive correlation. X-Tile plot of the patient age divided at the mean age, the optimal cut-point highlighted by the black circle in middle of the color bar. **B** Histogram of the entire cohort, optimal cut-off values of age at diagnosis were identified as 53 years based on cancer-specific survival (CSS). The X-axis is the age of patients, and the Y-axis is the number of patients at that age. **C** Kaplan-Meier survival curve developed based on these cutoff values

We obtained 45,974 CCRCC patients in totally. The median age of these patients was 60 years old (interquartile range: 51–69). At the same time, X tile result showed that 53 years old was defined as the best cut-off value for age (Fig. 1). Then we divided the cohort into two groups: younger group (53 years or younger), older group (older than 53 years) according to the cut-off value. The detailed features of the patients between the two groups were presented in Table 1.

**Table 1:** The Clinical characteristic baseline of CCRC patients in SEER

Characteristic	Before PSM			After PSM		
	Younger (<=53 y) N(%)	Older (> 53 y) N(%)	*P*	Younger (<=53 y) N(%)	Older (> 53 y) N(%)	*P*
**Sex**			< 0.001			0.613
Male	9134 (63.98)	18,958 (59.81)		9124 (63.96)	9165 (64.24)	
Female	5142 (36.02)	12,740 (40.19)		5142 (36.04)	5101 (35.76)	
**Race**			< 0.001			0.322
Black	1020 (7.14)	1939 (6.12)		1014 (7.11)	1063 (7.45)	
White	12,199 (85.45)	27,428 (86.53)		12,195 (85.48)	12,105 (84.85)	
Other	1057 (7.40)	2331 (7.35)		1057 (7.41)	1098 (7.70)	
**Marital**			< 0.001			0.739
Single/Other	5124 (35.89)	10,484 (33.07)		5116 (35.86)	5143 (36.05)	
Married	9152 (64.11)	21,214 (66.93)		9150 (64.14)	9123 (63.95)	
**Size**			< 0.001			0.571
Dmax<=4 cm	7058 (49.44)	12,966 (40.90)		7052 (49.43)	6981 (48.93)	
4 cm < Dmax<=7 cm	3771 (26.41)	10,175 (32.10)		3771 (26.43)	3847 (26.97)	
Dmax> 7 cm	3447 (24.15)	8557 (27)		3443 (24.13)	3438 (24.10)	
**Grade**			< 0.001			0.566
I	2204 (15.44)	4226 (13.33)		2199 (15.41)	2243 (15.72)	
II	7893 (55.29)	16,362 (51.62)		7890 (55.31)	7873 (55.19)	
III	3376 (23.65)	8978 (28.32)		3375 (23.66)	3309 (23.20)	
IV	803 (5.62)	2132 (6.73)		802 (5.62)	841 (5.90)	
**Laterality**			0.228			0.840
Left	6949 (48.68)	15,622 (49.28)		6946 (48.69)	6963 (48.81)	
Right	7327 (51.32)	16,076 (50.72)		7320 (51.31)	7303 (51.19)	
**SEER historic stage A**			< 0.001			0.754
Localized	11,405 (79.89)	21,988 (69.37)		11,395 (79.88)	11,345 (79.52)	
Regional	1865 (13.06)	6576 (20.75)		1865 (13.07)	1892 (13.26)	
Distant	1006 (7.05)	3134 (9.89)		1006 (7.05)	1029 (7.21)	
**Radiation**			0.035			0.202
NO	13,967 (97.84)	30,909 (97.51)		13,960 (97.86)	13,928 (97.63)	
YES	309 (2.16)	789 (2.49)		306 (2.14)	338 (2.37)	
**Chemotherapy**			< 0.001			0.274
NO	13,664 (95.71)	29,986 (94.60)		13,655 (95.72)	13,617 (95.45)	
YES	612 (4.29)	1712 (5.40)		611 (4.28)	649 (4.55)	

### Survival analysis

**Fig. 2:**
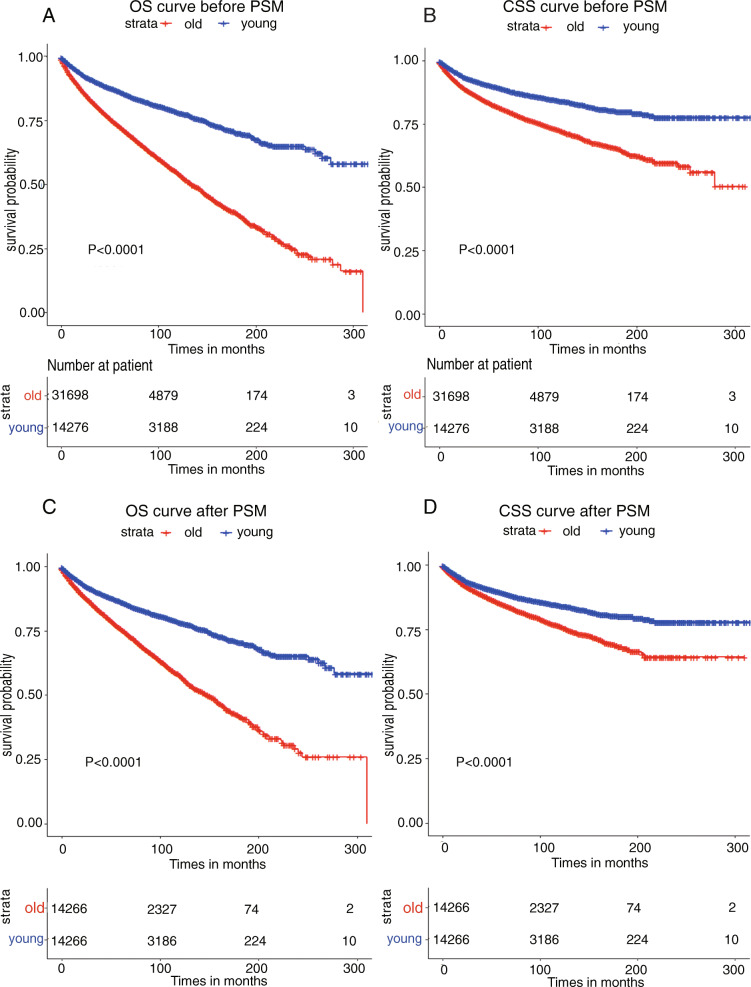
The overall survival (OS) and cancer-specific survival (CSS) curve of younger and older patients. **A** Kaplan-Meier survival curve of OS using unadjusted data in the Surveillance, Epidemiology, and End Results (SEER) data set. The 5- and 1-Year OS rates were 86.4 and 78.2%, respectively, and 72.8 and 54.5%, respectively (*P* < 0.001). **B** Kaplan-Meier survival curve of CSS Using unadjusted data in the SEER data set. The 5- and 10-Year OS rates were 89.4 and 81.7%, respectively, and 84.3 and 72.8%, respectively (*P* < 0.001). **C** Kaplan-Meier survival curve of OS using propensity score matching adjusted data set. The hazard ratios of OS using univariate analysis in the matched cohort were 2.056(95% CI,1.948–2.170; *P* < 0.001). **D** Kaplan-Meier survival curve of CSS using propensity score matching adjusted data set. The hazard ratios of OS using univariate analysis in the matched cohort were 1.496(95% CI,1.399–1.600; *P* < 0.001). The *P* values were calculated using the cox model

In the young group, 5- and 10-year OS rates were 86.4 and 78.2% respectively. In the old group, 5- and 10-year OS rates were 72.8% and 54.5 respectively (*P* < 0.001; Fig. 2 A). Univariate analysis results indicated that age, sex, race, marital status, size, grade, laterality, SEER historic stage A, radiation and chemotherapy,could predict patient suvival outcomes. Meanwhile, multivariate analysis showed that the age, sex, race, marital status, size, grade, laterality, SEER historic stage A, radiation and chemotherapy were independent prognostic factor for CCRCC OS (Table 2).

**Table 2:** Cox regression analysis of overall survival

Characteristic	Univariate cox regression before PSM		Multivariate cox regression before PSM		Multivariate cox regression after PSM	
	95%CI	*P*	95%CI	*P*	95%CI	*P*
**Age**						
Young(≤53 y)	Reference		Reference		Reference	
Old(> 53 y)	2.338 (2.228–2.453)	< 0.001	2.132 (2.031–2.238)	< 0.001	2.128 (2.015–2.247)	< 0.001
**Sex**						
Male	Reference		Reference		Reference	
Female	0.829 (0.789–0.862)	< 0.001	0.909 (0.873–0.947)	< 0.001	0.910 (0.859–0.964)	0.001
**Race**						
Black	Reference		Reference		Reference	
White	0.895 (0.831–0.964)	0.003	0.836 (0.776–0.901)	< 0.001	0.795 (0.722–0.876)	< 0.001
Other	0.798 (0.721–0.884)	0 < 0.001	0.716 (0.647–0.794)	< 0.001	0.682 (0.593–0.783)	< 0.001
**Marital**						
Single/Other	Reference		Reference		Reference	
Married	0.780 (0.751–0.811)	< 0.001	0.729 (0.701–0.758)	< 0.001	0.711 (0.674–0.750)	< 0.001
**Size**						
Dmax<=4 cm	Reference		Reference		Reference	
4 cm < Dmax<=7 cm	1.967 (1.868–2.070)	< 0.001	1.403 (1.330–1.480)	< 0.001	1.382 (1.284–1.488)	< 0.001
Dmax> 7 cm	4.084 (3.895–4.283)	< 0.001	1.849 (1.748–1.956)	< 0.001	1.965 (1.823–2.118)	< 0.001
**Grade**						
I	Reference		Reference		Reference	
II	1.151 (1.081–1.225)	< 0.001	0.966 (0.907–1.029)	0.283	0.988 (0.907–1.075)	0.773
III	2.225 (2.086–2.373)	< 0.001	1.213 (1.133–1.298)	< 0.001	1.287 (1.172–1.412)	< 0.001
IV	5.296 (4.911–5.712)	< 0.001	1.823 (1.681–1.978)	< 0.001	1.856 (1.661–2.075)	< 0.001
**Laterality**		< 0.001				
Left	Reference		Reference		Reference	
Right	0.936 (0.902–0.971)	< 0.001	0.953 (0.918–0.989)	0.012	0.947 (0.899–0.997)	0.038
**SEER historic stage A**						
**Localized**	Reference		Reference		Reference	
Regional	2.705 (2.585–2.831)	< 0.001	1.830 (1.740–1.925)	< 0.001	1.860 (1.727–2.003)	< 0.001
Distant	11.243 (10.734–11.776)	< 0.001	5.996 (5.631–6.383)	< 0.001	6.694 (6.115–7.329)	< 0.001
**Radiation**						
NO	Reference		Reference		Reference	
YES	6.150 (5.725–6.607)	< 0.001	1.340 (1.239–1.450)	< 0.001	1.319 (1.187–1.465)	< 0.001
**Chemotherapy**						
NO	Reference		Reference		Reference	
YES	5.959 (5.637–6.299)	< 0.001	1.196 (1.120–1.278)	< 0.001	1.254 (1.144–1.375)	< 0.001

In the young group, 5- and 10-year CSS rates were 89.4 and 81.7% respectively. In the old group, 5- and 10-year CSS rates were 84.3 and 72.8% respectively (*P* < 0.001; Fig. 2 B). The results of univariate analysis showed that age, sex, marital status, size, grade, laterality, SEER historic stage A, radiation and chemotherapy were associated with patient’s prognosis. Multivariate analysis showed that age, marital status, size, grade, SEER historic stage A, radiation and chemotherapy were independent prognostic factors for CCRCC CSS (Table 3).

**Table 3:** Cox regression analysis of cancer-specific survival

Characteristic	Univariate cox regression before PSM		Multivariate cox regression before PSM		Multivariate cox regression after PSM	
	95%CL	*P*	95%CL	*P*	95%CL	*P*
**Age**						
Young(≤53 y)	Reference		Reference		Reference	
Old(> 53 y)	1.834 (1.731–1.942)	< 0.001	1.549 (1.462–1.642)	< 0.001	1.573 (1.470–1.682)	< 0.001
**Sex**						
Male	Reference		Reference		Reference	
Female	0.781 (0.743–0.821)	< 0.001	1.008 (0.957–1.061)	0.769	1.023 (0.950–1.102)	0.549
**Race**						
Black	Reference		Reference		Reference	
White	0.969 (0.879–1.068)	0.525				
Other	0.924 (0.811–1.052)	0.233				
**Marital**						
Single/Other	Reference		Reference		Reference	
Married	0.911 (0.866–0.957)	< 0.001	0.843 (0.801–.887)	< 0.001	0.824 (0.769–0.884)	< 0.001
**Size**						
Dmax<=4 cm	Reference		Reference		Reference	
4 cm < Dmax<=7 cm	3.886 (3.566–4.233)	< 0.001	2.324 (2.127–2.540)	< 0.001	2.433 (2.153–2.750)	< 0.001
Dmax> 7 cm	11.792 (10.896–12.762)	< 0.001	3.710 (3.393–4.055)	< 0.001	4.299 (3.814–4.845)	< 0.001
**Grade**						
I	Reference		Reference		Reference	
II	1.420 (1.287–1.567)	< 0.001	1.028 (0.931–1.135)	0.583	1.051 (0.918–1.203)	0.472
III	4.020 (3.649–4.428)	< 0.001	1.514 (1.369–1.675)	< 0.001	1.615 (1.405–1.855)	< 0.001
IV	10.814 (9.738–12.009)	< 0.001	2.229 (1.995–2.490)	< 0.001	2.264 (1.944–2.636)	< 0.001
**Laterality**						
Left	Reference		Reference		Reference	
Right	0.931 (0.887–0.976)	0.003	0.954 (0.910–1.001)	0.054	0.940 (0.880–1.005)	0.07
**SEER historic stage A**						
Localized	Reference		Reference		Reference	
Regional	4.934 (4.637–5.249)	< 0.001	2.615 (2.443–2.798)	< 0.001	2.641 (2.402–2.903)	< 0.001
Distant	25.576 (24.104–27. 138)	< 0.001	9.900 (9.172–10.685)	< 0.001	10.134 (9.108–11.276)	< 0.001
**Radiation**						
NO	Reference		Reference		Reference	
YES	9.242 (8.574–9.962)	< 0.001	1.446 (1.334–1.568)	< 0.001	1.458 (1.309–1.625)	< 0.001
**chemotherapy**						
NO	Reference		Reference		Reference	
YES	8.887 (8.369–9.438)	< 0.001	1.179 (1.100–1.263)	< 0.001	1.268 (1.152–1.396)	< 0.001

### Survival analysis after PSM

The clinical characteristics of the patients between the younger and older groups had obvious differences. So PSM method was applied to balance the differences between the variables, and generated a new queue (All covariates were well balanced, *P* values > 0.05; Table 1). Univariate analysis results showed that HR for OS of the older patient were 2.056 (95% CI:1.948–2.170; *P* < 0.001), HR for CSS were 1.496(95% CI, 1.399–1.600; *P* < 0.001) when compared with the younger group. In the PSM queue, the younger people also had a higher survival rate than older people (Fig. 2 C, D). Multivariate analysis results showed that compared with the younger group, HR for OS of the older patient were 2.128(95%CI:2.015–2.247; *P* < 0.001), HR for CSS were 1.573(95%CI:1.470–1.682; *P* < 0.001). Other variable results were showed in Tables 2, and 3.

### Subgroup analysis

**Fig. 3:**
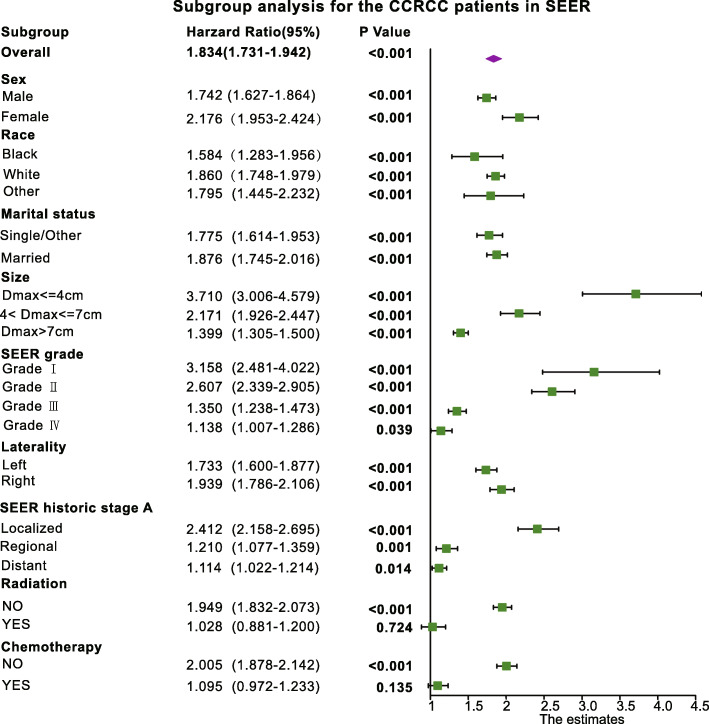
The subgroup analyses were performed according to age (Young group vs. Older group) of CCRC patients in SEER data set. According to the patients’ sex, race, marital status, size, grade, laterality, SEER historic stage A, radiation, and chemotherapy, we divide CCRCC patients into different subgroups such as male, female, black, white, other race, single/other, married, tumor size dmax<=4 cm, 4 cm < dmax<=7 cm, grade I, II, III, IV, left, rught, Localized, regional, distant, radiation no, radiation yes, chemothera no, chemothera yes, and compare the survival difference between young and old in each subgroup and calculate the corresponding HR value

We performed a subgroup analysis based on sex, race, marital status, size, grade, laterality, SEER historic stage A, radiation, and chemotherapy. In most subgroups, the older group had a worse prognosis than the younger group. However radiation, and chemotherapy and prognostic differences between young and old groups were not statistically significant (*P* > 0.05) (Fig. 3).

### Differential expressed genes and prognosis related genes

**Fig. 4:**
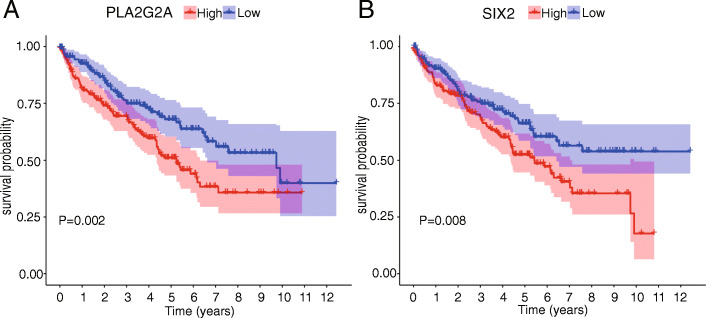
Survival analysis of DEGs. **A** Kaplan-Meier analysis were developed based on expression level of PLA2G2A (*p* = 0.002); **B** Kaplan-Meier analysis were developed based on expression level of SIX2 (*p* = 0.008)

The RNA-seq data of 530 CCRCC samples were downloaded from TCGA database. According to the cut-off value of 53 years old, they were divided into 158 young group and 372 elderly group. We finally got 11 differential expressed genes (DEGs) between the younger and older groups in CCRCC (Table 4). Among them, SIX2, THBS4 and PLA2G2A were up regulated in elderly patients with CCRCC. NKX2–3, CD1A, SCUBE1, NEFH, MYL10, TBL1Y, DYTN and SLC4A10 were down regulated in elderly patients with CCRCC. Then, the DEGs were analyzed by survival. The results showed that high expression of SIX2 and PLA2G2A were associated with poor prognosis in the elderly (Fig. 4).

**Table 4:** The 11 age-related differentially expressed genes

Gene	Young group Mean	Older group Mean	LogFC	*P* Value	FDR
NKX2–3	0.248444555	0.090005562	−1.464837864	0.000165397	0.037107612
PLA2G2A	0.538674035	1.300683714	1.271785754	0.00040971	0.045159714
CD1A	0.499103859	0.182836665	−1.448784648	2.71E-05	0.018220037
SCUBE1	0.90569885	0.448615793	−1.013551014	2.35E-05	0.018220037
NEFH	0.536764531	0.234581834	−1.194198049	2.75E-06	0.006374551
DYTN	0.029518103	0.006416315	−2.201783071	4.53E-05	0.019711439
SIX2	0.240527498	0.499819682	1.055205878	0.000307207	0.043814576
TBL1Y	0.119504132	0.055953453	−1.094761418	1.76E-05	0.017134562
MYL10	0.058554689	0.024582676	−1.252142696	0.000530706	0.047321306
SLC4A10	0.111875634	0.049159421	−1.186356037	3.25E-05	0.018220037
THBS4	1.421111755	3.163220721	1.154374215	3.38E-05	0.018220037

## Discussion

A total of 45,974 CCRCC patients were included in the SEER database, of which the 53-year-old cut-off value was used to divide the younger and older groups. Survival analysis results showed that younger age (under age 53) was an independent predictor of CCRCC. And we obtained some genes related to old patients with CCRCC by analyzing the RNA-seq data downloaded from TCGA.

Some studies reported that the 40 years old was suitable to act as the dividing line between young and old CCRCC patients. Xavier Taccoen et al. found that young (under 40 years of age) age was an independent prognostic factor for CCRCC, with a better prognosis [8]. Atiqullah Aziz et al. found that young patients with RCC (age 40 or under) have a significantly lower all cause and disease specific killed [9]. Ho Won Kang et al. also found that young age was associated with favorable pathological features, although it is not survival independent prognostic factors in surgically treated RCC patients. But the result of the Kaplan-Meier analysis showed that the CCS rate was significantly better in the young age group than the other groups (middle age: ≥ 4 and < 60 years; old age: ≥ 60 years) [10]. Analysis results between younger and older RCC patients (20–39 and 40–79) of Jeong Ho Kim et al. found that younger RCC patients would have more favorable histological subtypes. And the 5-year CSS rates for young and older patients were 95.5 and 90.5% respectively. However, after PSM, the five-year CSS rate was 95.5% for the younger group and 94.7% for the older group, and the prognosis was not significantly different (log rank *p* = 0.184) [11]. In addition to the 40 years old, there were many age cut-off values, such as 45 and 55 years old. Yoshinobu Komai et al. used the 45-year-old as a cut-off value for younger and older group. Compared with the older patients, the young patients with RCC had similar recurrence-free survival rates but better CSS rates [12]. Eun-Jung Jung et al. believed that younger age was an independent predictor of prognosis through multivariate analysis. Whereas in their study, younger than 55 years of age was considered as young in CCRCC [13]. In this study, we used X-tile plots to assessed all possible age cut-off value, and finally selected the age 53 as the cut off value for dividing younger and older group. And younger groups had better OS and CSS compared to older groups. What’s more, in the subgroup analysis, the prognosis of old group was worse than that of young group in all subgroups of this study, especially in the Dmax <= 4 cm subgroups (HR = 3.710(3.006–4.579), *P* < 0.001). It suggests that in future CCRCC clinical decision-making, patients older than 53 years old needed to pay more attention and better treatment options. Compared with younger people, older patients have a greater risk of worsening disease, lower survival rate, and worse treatment efficiency, which may be related to the physical fitness of the older patients and probably diseases that may existed in themselves.

In recent years, with the development of cancer gene sequencing and targeted therapies, the research on gene expression of CCRCC had made some progress. In the CCRCC age-related studies, Xp11 translocation renal cell carcinoma was kind of RCC subtype, Malouf GG et al. used the targeted therapy to treat patients, the objective responses was achieved and the patients got the better progression-free survival [14]. Mitchell TJ et al. analyzed the entire genome of CCRCC and found that 36% of patients experience 3p loss and 5q gain, which usually occured during childhood or adolescence. Meanwhile hotspots of point mutations in the 5′ UTR of TERT, targeting a MYC-MAX-MAD1 repressor associated with telomere lengthening [15]. Malouf GG reported that ASPSCR1-TFE3 might be the most aggressive among the transcription factor E3 fusion genes in RCC patients [16].

In this study, we obtained 11 DEGs by comparing RNA-seq data from younger and older CCRCC patients. Then, the DEGs were used for survival analysis. As showed in the result (Fig. 4), the expression of Secretory Phospholipase A2 Group IIA (PLA2G2A), and Sine oculis-related homeobox 2(SIX2) were related to the survival of the elderly. Secretory Phospholipase A2 Group IIA (PLA2G2A), one of the family members of PLA2, primarily targets extracellular phospholipids with implications in host antimicrobial defense, inflammatory response and tissue regeneration [17]. And PLA2G2A was found to be associated with different disease states including cancer. Our results indicate that PLA2G2A is highly expressed in the elderly and is closely related to poor prognosis in the elderly group. However, further studies are needed to illuminate the molecular and biological mechanism of PLA2G2A in CCRCC. Sine oculis-related homeobox 2 (SIX2) is composed of six homeobox genes (SIX1-SIX6), which serves as an important regulator of embryonic development. Wu Y et al. [18] found that overexpression of Six 2 increased the proliferative capacity of cells and decreased apoptosis in clear cell renal cell carcinoma. At the same time, our research showed that SIX2 was age-related DEG. And high expression levels of SIX2 was related to poor prognosis of the elderly. These results suggest that PLA2G2A and SIX2 might have clinical monitoring value in CCRCC which deserved for further research.

Our study had several potential limitations. The leading known risk factors for renal cancer were smoking, obesity and hypertension [19–24]. However, due to the lack of corresponding data in the SEER database, we were unable to study these factors. At the same time, retrospective analyses always carried the risk of various biases. We used the subgroup, PSM analysis and incorporate large amounts of patients in this study to minimize potential biases.

## Conclusions

In conclusion, we proposed that 53-year-old was a reasonable cut-off value among CCRCC patients, and the elderly group had a worse prognosis than the younger group. These results remind clinicians that more attention and better treatment should be given to CCRCC patients older than 53 years old. At the same time, 11 gene were age-related differential genes. The high expression of PLA2G2A and SIX2 might be associated with poor prognosis in the elderly, but the specific mechanism remained to be further studied.

## Data Availability

The datasets analyzed during the current study download from Surveillance Epidemiology and End Results database (https://seer.cancer.gov/) and The Cancer Genome Atlas (TCGA) database. (https://cancergenome.nih.gov/).

## Abbreviations

CCRCC: Clear cell renal cell carcinoma
DEGs: Differentially expressed genes
SEER: Surveillance Epidemiology and End Results
TCGA: The Cancer Genome Atlas
OS: Overall survival.
CSS: Cancer specific survival
PSM: Propensity score matching
HR: Hazard Ratio
FC: Fold change
FDR: False discovery rate
CPM: Counts per million
